# Duck plague virus UL24 protein initiates K48/K63-linked IRF7 polyubiquitination to antagonize the innate immune response

**DOI:** 10.1016/j.psj.2024.104378

**Published:** 2024-10-04

**Authors:** Peilin Ruan, Yalin Chen, Mingshu Wang, Anchun Cheng, Qiao Yang, Bin Tian, Xumin Ou, Di Sun, Yu He, Zhen Wu, Juan Huang, Ying Wu, Shaqiu Zhang, Xinxin Zhao, Dekang Zhu, Renyong Jia, Mafeng Liu, Shun Chen

**Affiliations:** ⁎ College of Animal Science and Veterinary Medicine, Institute of Veterinary Medicine and Immunology, GuiZhou University, GuiYang 550025, China; † Key Laboratory of Animal Disease and Human Health of Sichuan Province, Chengdu 611130, China; ‡ International Joint Research Center for Animal Disease Prevention and Control of Sichuan Province, Chengdu 611130, China; § College of Veterinary Medicine, Institute of Veterinary Medicine and Immunology, Sichuan Agricultural University, Chengdu 611130, China; ║ Research Center of Avian Disease, College of Veterinary Medicine, Sichuan Agricultural University, Chengdu 611130, China; ¶ Engineering Research Center of Southwest Animal Disease Prevention and Control Technology, Ministry of Education of the People's Republic of China, Chengdu 611130, China

**Keywords:** Duck plague virus, UL24, cGAS-STING, proteasome pathway, innate immunity

## Abstract

Duck plague virus (**DPV**), which is the causative agent of duck viral enteritis, is highly infectious and can cause severe disease and death in ducks, geese and other waterfowl. Several tegument proteins of DPV have been shown to affect the cyclic GMP-AMP synthase (cGAS)-STING signaling pathway to modulate host innate immune responses. DPV UL24, an important DPV tegument protein, can inhibit the activity of the IFN-β promoter. However, the mechanism by which the DPV UL24 protein regulates the host innate immune response remains unclear. In this study, we found that the UL24 protein can significantly inhibit the activity of the interferon-β promoter induced by poly(I:C) and reduce the production of IFN-β, interferon-stimulated genes (OASL, Mx), and the cellular inflammatory factor IL-6. 2) The UL24 protein can widely inhibit the mRNA level of immune signaling molecules. The UL24 protein can also downregulate the protein expression of RIG-I, MDA5, MAVS, cGAS, STING, TBK1 and IRF7 in DEFs. RT-qPCR results revealed that UL24 significantly inhibited the mRNA accumulation for the immune signaling molecules cGAS, STING, TBK1 and IRF7. 3) The UL24 protein induced the degradation of IRF7 via ubiquitination. After the DEFs were treated with the ubiquitin proteasome inhibitor MG132, the degradation of IRF7 by the UL24 protein was alleviated. Coimmunoprecipitation results revealed that DPV UL24 induced the K48/K63-linked ubiquitination of IRF7, which promoted its degradation and thus antagonized the host innate immune response.

## INTRODUCTION

Duck viral enteritis, which is also known as duck plague (**DP**), is an acute, febrile and septicemic infectious disease that affects ducks, geese and many types of wild, goose-like animals and is caused by duck plague virus (**DPV**) (Guo et al., 2009; Ruan et al., 2022). DPV, also known as Anatid herpesvirus 1 (**AHV-1**), is a spherical virus with an envelope and measures approximately 160∼180 nm in diameter; DPV consists of four parts: the core, capsid, tegument and envelope. DPV is a double-stranded DNA virus that belongs to the Herpesviridae family, the alpha-herpesvirus subfamily, and Marek's virus genus (Wu et al., 2012a). The DPV genome is approximately 162 kb in length and contains 78 open reading frames (**ORFs**), including 65 ORFs in a unique long region (**UL**) and 11 ORFs in a unique short region (**US**); the remaining 2 ORFs are located in intermediate repeats (**IRSs**) and terminal repeats (**TRSs**) (Wu et al., 2012b; Wu et al., 2017; Shen et al., 2023; Zhou et al., 2023). Duck viral enteritis was first reported in 1923. Currently, this disease frequently occurs in waterfowl breeding areas worldwide (Dhama et al., 2017). Moreover, duck viral enteritis has damaged the duck industry for a long time, causing great economic losses in this industry globally. In recent years, some progress has been made in research on the pathogenesis of DPV, but the mechanism underlying DPV resistance to host innate immunity requires further study.

Innate immunity is an important defense mechanism for a host that is infected by a pathogen (O'Connor and Sen, 2021). Following viral infection, pattern recognition receptors (**PRR**) in host cells recognize viral pathogen-associated molecular patterns (PAMPs), activate complex innate immune signaling pathways, and induce the expression of type I interferons, proinflammatory cytokines and other downstream antiviral effector proteins that inhibit viral replication and clear the virus from the body (Baccala et al., 2009; Tan et al., 2018). Common PRRs include Toll-like receptors (**TLR**), NOD-like receptors (**NLR**), RIG-I-like receptors (**RLR**) and DNA-related recognition receptors (Lee and Kim, 2007; Hornung et al., 2006). Cyclic GMP-AMP synthase (cGAS) is a cytosolic DNA PRR that activates the cGAS-STING pathway to mediate innate immune responses after viral DNA is sensed (Hopfner and Hornung, 2020). Recent studies have shown that herpes simplex virus type 1 (**HSV-1**), pseudorabies virus (**PRV**), Marek's disease virus (**MDV**) and DPV can antagonize host innate immunity by blocking the cGAS-STING signaling pathway (Huang et al., 2018; Kong et al., 2022; Li et al., 2019; Li et al., 2020; Gao et al., 2022).

**Figure 1:**
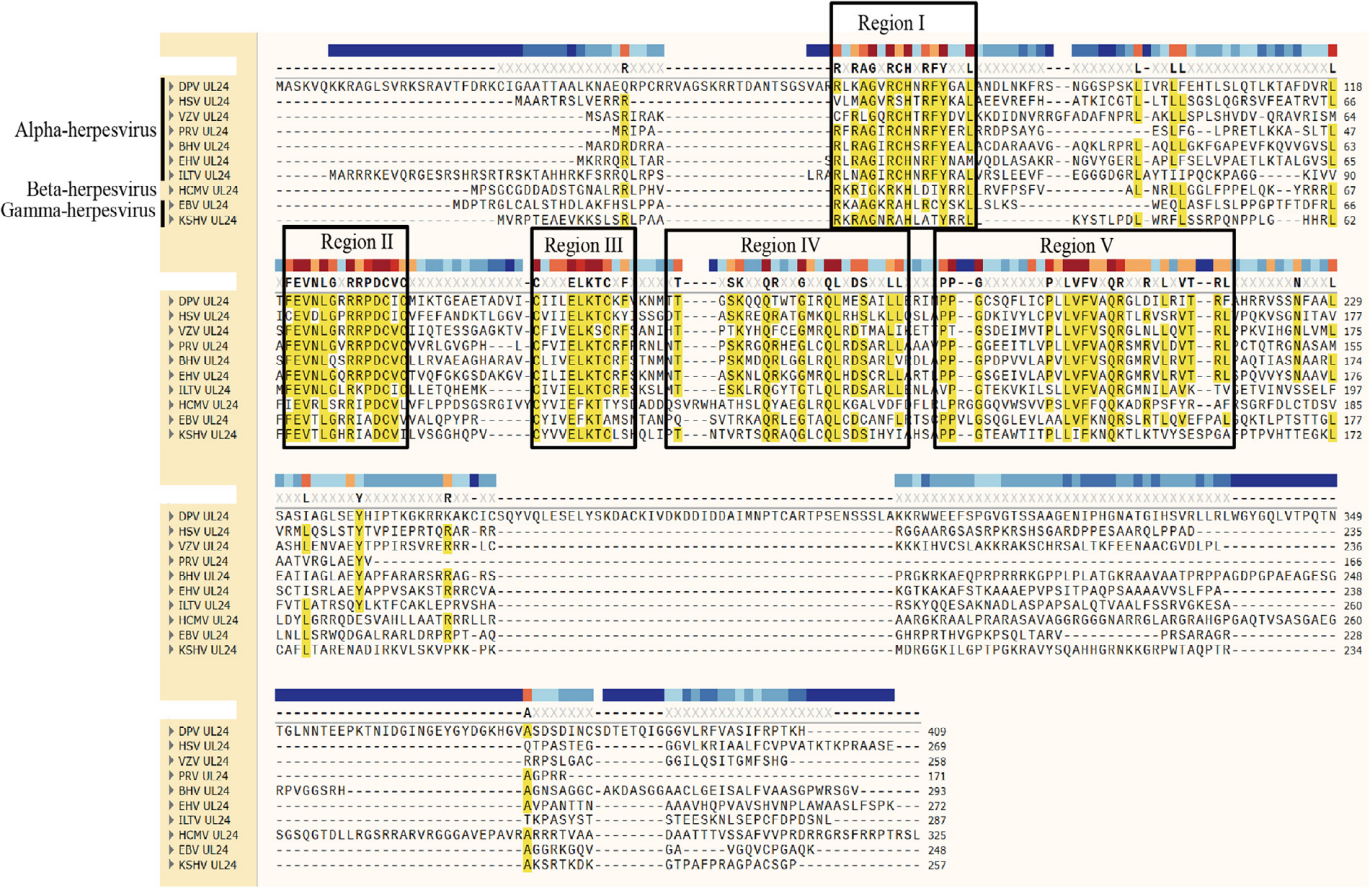
Comparison of herpesvirus UL24 protein homology. The homology of DPV UL24 was compared with that of HSV-1 UL24 (YP_009137098.1), VZV UL24 (NP_040158.1), PRV UL24 (YP_010795426.1), BHV UL24 (UWL63377.1), EHV UL24 (YP_053083.1), and ILTV UL24 (YP_010795253.1) in Alphaherpesviridae; HCMV UL24 (YP_081524.1) in Betaherpesviridae; and EBV UL24 (YP_401702.1) and KSHV UL24 (YP_001129373.1) in Gammaherpesviridae.

Among the members of the herpesvirus family, the UL24 gene is located in a unique long region. In addition to the UL76 gene of human cytomegalovirus (**HCMV**), the UL24 and UL23 genes of other herpesviruses are arranged in a head-to-head manner at the 5′ end (Jacobson et al., 1989, Pearson and Coen, 2002, Ruan et al., 2023). UL24 is considered a core gene of herpesviruses and is present in all Herpesviridae members (Lymberopoulos and Pearson, 2007, Bertrand et al., 2010, Carvalho et al., 2012, Gao et al., 2017). In herpesviruses, the UL24 protein consists of five highly conserved functional domains that determine most of its functions and are important for the life cycle of the virus (Figure 1) (Ruan et al., 2023). Therefore, we speculate that UL24 homologs of different herpesviruses have similar functions. The mechanism by which the HSV-1 and PRV UL24 proteins inhibit innate immunity has been described. HSV-1 UL24 reduces the nuclear translocation of p65 and p50, and PRV UL24 negatively regulates ISG20 transcription (Ruan et al., 2023, Xu et al., 2017, Chen et al., 2021). DPV UL41 encodes the host-closing protein VHS to reduce the expression of IRF7, and DPV Us3 phosphorylates IRF7 to antagonize the host's innate immune response (Gao et al., 2022, Liu et al., 2022). DPV UL24 can inhibit IFN-β promoter activity to antagonize the host's innate immune response, but its detailed mechanism remains to be explored. In this study, we found that DPV UL24 significantly downregulated IFN-β activity and inhibited the expression of nodal proteins in the cGAS-STING and RIG-I/MDA5-MAVS signaling pathways; furthermore, the UL24 protein promoted the degradation of IRF7 through the ubiquitin-proteasome pathway. These findings provide new insights into the mechanisms by which DPV UL24 antagonizes host innate immunity.

## MATERIALS AND METHODS

### Cells, Reagents and Antibodies

DEFs were prepared from 9-day-old duck embryos and cultured in DMEM (Gibco, Shanghai, China) supplemented with 10% newborn bovine serum (**NBS**) in 5% CO_2_ at 37°C. Dual-luciferase reagent was purchased from Promega (Madison, WI, USA), poly(I:C) was obtained from Sigma-Aldrich, and the proteasome inhibitor MG132 and the autophagy lysosomal inhibitor CQ were obtained from Selleck. Commercial rabbit anti-Flag, mouse anti-HA, mouse anti-Myc, mouse anti-GAPDH, and goat anti-mouse and goat anti-rabbit antibodies were purchased from Proteintech (Wuhan, China).

### Plasmids

The DH5α strain and eukaryotic expression plasmids pCAGGS, pCAGGS-3Flag, pCAGGS-3HA, cGAS-HA, STING-Flag, STING-Myc, TBK1-Flag, IRF7-Myc, RIG-I-Flag, MDA5-Flag, MAVS-Flag, UL2-3HA IFN-β-Luc, pRL-TK, HA-Ub and its mutant plasmids were preserved and provided by the Avian Disease Prevention and Control Research Center of Sichuan Agricultural University. The UL24 fragment was amplified by PCR and inserted into the pCAGGS-3Flag and pCAGGS-3HA vectors to generate the UL24-3Flag and UL24-3HA plasmids used in this study. The primers used in this study are shown in Table 1.

**Table 1:** Sequence and characteristics of the PCR/q-PCR primers.

Primers	Primer sequence (5′–3′)	Gene
UL24-3Flag-F	CATCATTTTGGCAAAGAATTCGCCACCATGGCATCGAAGGTACAGAA	UL24-3Flag
UL24-3Flag-R	GTCGTCATCCTTGTAATCGGTACCGTGTTTAGTTGGTCTGAATA	
UL24-3HA-F	CATCATTTTGGCAAAGAATTCGCCACCATGGCATCGAAGGTACAGAA	UL24-3HA
UL24-3HA-R	TGCATAATCCGGCACATCATAAGGATAGGTACCGTGTTTAGTTGGTCTGAATA	
cGAS-F	CTACTACGAGCGCGTCAAGA	*cGAS*
cGAS-R	CTGAATCCTCGCGATAGGCA	
STING-F	GAGATGACCGAGAGGTCCCA	*STING*
STING-R	ACACTCCTTTATGCGTGGCA	
TBK1-F	TTAGAGGAGCCATCCAACGC	*TBK1*
TBK1-R	AGTTCTCTCGCAGCACCAAA	
IRF7-F	AACGCCAGGAAGGATGTCAC	*IRF7*
IRF7-R	CGCAGCGAAAGTTGGTCTTC	
IFN-β-F	TCTACAGAGCCTTGCCTGCAT	*IFN-β*
IFN-β-R	TGTCGGTGTCCAAAAGGATGT	
OASL-F	TCTTCCTCAGCTGCTTCTCC	*OASL*
OASL-R	ACTTCGATGGACTCGCTGTT	
Mx-F	TGCTGTCCTTCATGACTTCG	*Mx*
Mx-R	GCTTTGCTGAGCCGATTAAC	
IL-6-F	TTCGACGAGGAGAAATGCTT	*IL-6*
IL-6-R	CCTTATCGTCGTTGCCAGAT	
18s RNA-F	GTACAGTGAAACTGCGAATGG	*18 sRNA*
18s RNA-R	CGTCGGCATGTATTAGCTCTA	

### Dual Luciferase Reporter Assay

The indicated eukaryotic expression plasmid was transfected into DEFs together with the firefly luciferase reporter plasmid IFN-β-luc using Hieff TransTM reagent according to the manufacturer's instructions (Yeasen Corporation), and the Renilla luciferase reporter gene pRL-TK plasmid was used as an internal control. At 36 h after transfection, the cells were lysed to obtain samples, which were assayed using a dual luciferase reporter assay system (Promega Corporation). Three biological replicates were used for all the samples, and each sample was tested three times.

### Real-Time qPCR

Total RNA was extracted, and reverse transcription was performed using the Prime Script RT reagent Kit (Takara, Japan). The expression level of each cDNA was determined by real-time qPCR. The specific quantitative primers used to analyze cGAS, STING, TBK1, IRF7, IFN-β, OASL, Mx and IL-6 expression are shown in Table 1, and the duck 18S RNA gene was used as an internal reference. The reaction mixture included 5 μL of SYBR, 0.3 μL of forward and reverse primers, 1 μL of cDNA and 3.4 μL of deionized water. The qPCR procedure was as follows: initial denaturation at 95°C for 1 min, followed by 40 cycles of 95°C denaturation for 15 s and 60°C extension for 1 min. The resulting qPCR products were quantified by comparing them with the established standard curve of the laboratory. Three biological replicates were used for all the samples. All the reactions were performed in triplicate, and at least three independent experiments were performed.

### Coimmunoprecipitation and Western Blotting

A eukaryotic expression plasmid was transfected into DEFs. After 36 h, the cells were placed on ice, after which the IP solution was added (Beyotime, Beijing, China). The supernatants were centrifuged, incubated with the corresponding antibody overnight, and incubated with magnetic beads (Beyotime, Beijing, China) at room temperature for 1 h. The resulting cell lysates were separated using SDS-PAGE, and the proteins were subsequently transferred to polyvinylidene difluoride (**PVDF**) membranes (Millipore, MA, USA), which were subsequently blocked with blocking buffer (5% skim milk and 0.1% Tween 20 in PBS) for 2 h at room temperature. The membranes were incubated with primary and secondary antibodies corresponding to the target proteins. The membranes were washed three times with PBST, and the signals were visualized using an enhanced chemiluminescence (**ECL**) kit (Takara, Japan).

### Indirect Immunofluorescence Assay

UL24-3Flag and IRF7-Myc were transfected into DEFs on slides according to the corresponding experimental groups. After 36 h, the cells were fixed with 4% paraformaldehyde overnight, permeabilized with 0.25% Triton X-100 at 4°C for 30 min, and blocked with 5% BSA overnight. Rabbit anti-Flag (1:1000) and mouse anti-Myc (1:1000) antibodies were added and incubated overnight at 4°C, and Alexa Fluor-conjugated secondary antibodies (1:1000) in 1% PBST-bovine serum albumin (BSA) buffer were added and incubated at 37°C for 60 min. We examined the samples under a Nikon H550 L fluorescence microscope.

### Inhibition of Protein Degradation Pathway

DEFs were co-transfected with IRF7-Myc, UL24-3Flag or pCAGGS and cultured for 24 h. The cells were treated with the proteasome inhibitor MG-132 or the lysosomal inhibitor CQ for 12 h. Cell samples were collected, and the protein expression of IRF7 was detected by Western Blotting.

### Statistical Analysis

All the data were analyzed by 1-way ANOVA with GraphPad Prism 8.0. The *p* values are indicated by asterisks; ns indicates not significant (*P* > 0.05), * indicates *P* ≤ 0.05, ** indicates *P* ≤ 0.01, and ** indicates *P* ≤ 0.001. *P* ≤ 0.05 was considered significant.

## RESULTS

### DPV UL24 Inhibits the Signaling Pathway Upstream of IFN-β

**Figure 2:**
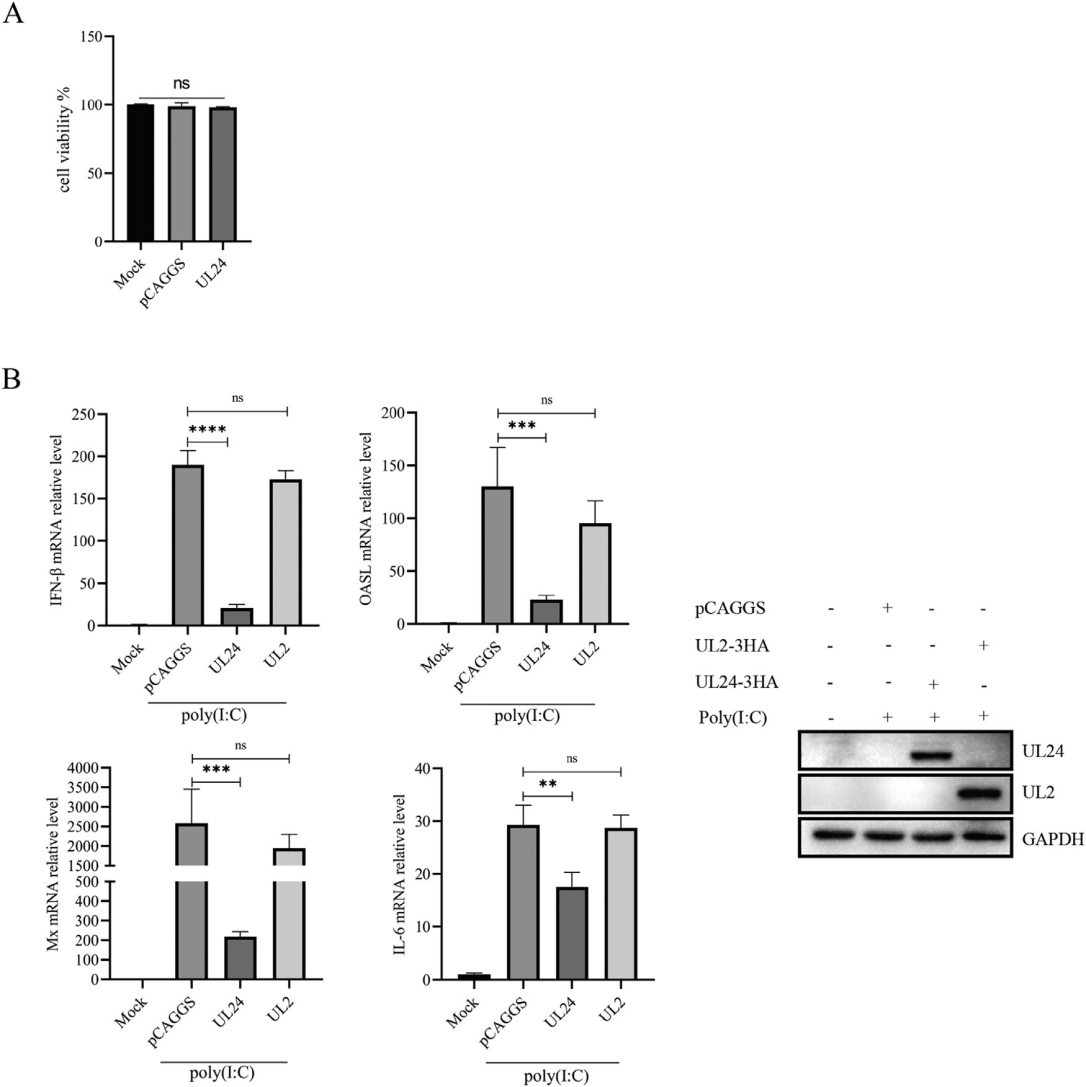
DPV UL24 inhibits the upstream IFN-β signaling pathway. (A) DEFs were transfected with the UL24-3Flag or empty vector. Cell viability was determined with a Cell Counting Kit (CCK-8) after 36 h. (B) DEFs were transfected with UL24-3HA, UL2-3HA or empty vector for 24 h and then transfected with poly (I:C) for 12 h; the mRNA levels of IFN-β, IL-6, Mx, and OASL were measured by qPCR. UL24 and UL2 expression was examined by Western blotting at 12 h after poly (I:C) transfection. (ns, *P* > 0.05; *, *P* ≤ 0.05; **, *P* ≤ 0.01; ***, *P* ≤ 0.001; ****; *P* ≤ 0.0001; and 1s-way ANOVA).

Poly (I:C) can activate immune signaling pathways in duck embryo fibroblasts (**DEF**) and subsequently upregulate the transcription and expression of IFN-β as well as the IFN-stimulated genes OASL, Mx and IL-6. In this study, we used the poly (I:C) stimulation of DEFs to activate the signaling pathway that leads to IFN-β production, and we measured the mRNA expression of related cytokines in cells that were transfected with UL24-3HA, UL2-3HA or an empty vector. First, we confirmed that overexpression of the UL24 protein does not cause toxicity to cells (Figure 2A). The UL24 protein significantly inhibited the poly (I:C)-induced mRNA expression of IFN-β, OASL, Mx and IL-6, but another viral protein, UL2, did not significantly inhibit the transcription of these immune factors (Figure 2B). These results suggest that DPV UL24 can specifically downregulate the expression of immune factors in host cells and suppress the innate immune response of host cells.

### DPV UL24 Inhibits the Activation of IFN-β in Host Cells

**Figure 3:**
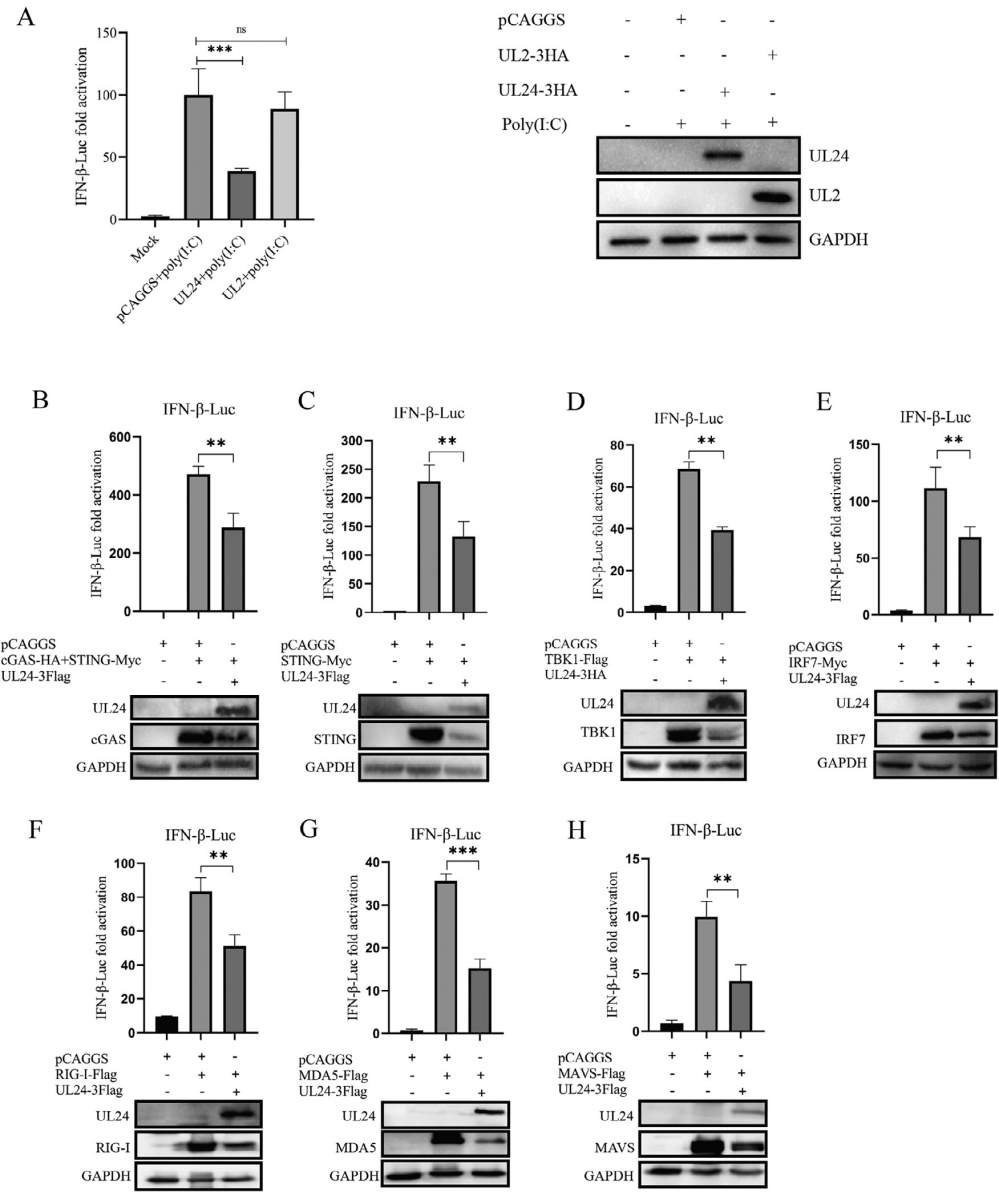
DPV UL 24 inhibits the activation of IFN-β in host cells. (A) After pCAGGS, UL24 or UL2 plasmids were transfected into DEFs for 24 h, poly (I:C) was used to activate the immune response of DEFs, and the promoter activity of IFN-β was analyzed with a dual-luciferase reporter assay. (B-H) The UL24 protein was co-expressed with cGAS, STING, TBK1, IRF7, RIG-I, MDA5 and MAVS in DEFs to determine the effect of the UL24 protein on IFN-β promoter activity. (ns, *P* > 0.05; **; *P* ≤ 0.01; ***, *P* ≤ 0.001; and 1s-way ANOVA).

Intracellular PRRs are key receptors that induce the production of type I interferons in cells infected with DPV; thus, DPV UL24 may inhibit PRR-mediated immune pathway signaling and thus inhibit IFN-β activation. We next asked whether DPV UL24 inhibits cGAS-, RIG-I- and MDA5-mediated IFN-β activation. We first examined the effect of the UL24 protein on IFN-β promoter activity and subsequently further examined the effect of the UL24 protein on DNA recognition signaling pathway- and RNA recognition signaling pathway-mediated IFN-β activation. A dual-luciferase reporter gene assay revealed that the UL24 protein significantly decreased IFN-β promoter activity, whereas UL2 had no inhibitory effect on IFN-β promoter activity (Figure 3A). When the UL24 protein was co-expressed with RIG-I, MDA5, MAVS and the node proteins of the cGAS-STING pathway in DEFs, IFN-β promoter activity was detected. The results revealed that the ability of cGAS-STING signaling pathway-related proteins to activate the IFN-β promoter was inhibited by the UL24 protein (Figure 3B–3E). The activation of the IFN-β promoter by RIG-I, MDA5 and their subordinate adaptor protein MAVS was also inhibited by DPV UL24 (Figure 3F–3H). Western blotting analysis also revealed that the UL24 protein downregulated the expression of RIG-I, MDA5, MAVS, cGAS, STING, TBK1 and IRF7 (Figure 3B–3H). Collectively, these results demonstrated that DPV UL24 antagonizes innate immune responses mediated by both the cGAS-STING signaling pathway and the RIG-I/MDA5-MAVS signaling pathway.

### DPV UL24 Inhibits the Expression of cGAS, STING, TBK1 and IRF7

**Figure 4:**
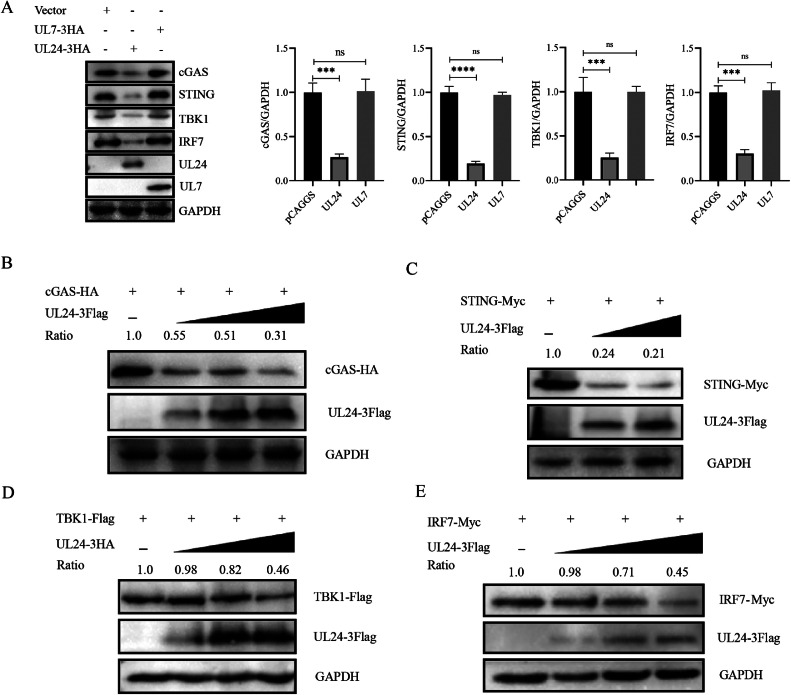
DPV UL 24 inhibits the expression of cGAS, STING, TBK1 and IRF7. (A) UL24-3Flag or UL7-3Flag plasmids or empty vectors were co-transfected with cGAS, STING, TBK1 or IRF7 into DEFs. Thirty-six hours after transfection, the cells were harvested and subjected to Western blotting analysis with the indicated antibodies. (B-E) The UL24 protein was co-expressed to different degrees with the host proteins cGAS, STING, TBK1 and IRF7 in DEFs, and 36 hours after transfection, its protein expression was measured. The “ratio” represents the ratio of the gray value of the host protein to that of the internal reference protein GAPDH (**, *P* ≤ 0.01; ***; *P* ≤ 0.001 ****, *P* ≤ 0.0001; and 1-way ANOVA).

DPV UL24 inhibited the activation of IFN-β by cGAS, STING, TBK1 and IRF7. We further investigated the effects of UL24 on the expression of these four proteins. pCAGGS, UL24-3HA and UL7-3HA were co-transfected with cGAS, STING, TBK1 and IRF7 eukaryotic expression plasmids, respectively, into DEFs. The results revealed that the UL24 protein significantly inhibited their expression, whereas the UL7 protein did not downregulate their expression (Figure 4A). cGAS-HA (400 ng), STING-Myc, TBK1-Flag, IRF7-Myc and UL24-3Flag (0ng, 200ng, 400ng, 600ng), pCAGGS (600ng, 400ng, 200ng, 0ng) were co-transfected into DEFs. The WB results revealed that the expression of cGAS, STING, TBK1 and IRF7 decreased gradually with the increasing UL24 protein expression (Figure 4B–4E).

### DPV UL24 Downregulated the Transcription of cGAS, STING, TBK1 and IRF7

**Figure 5:**
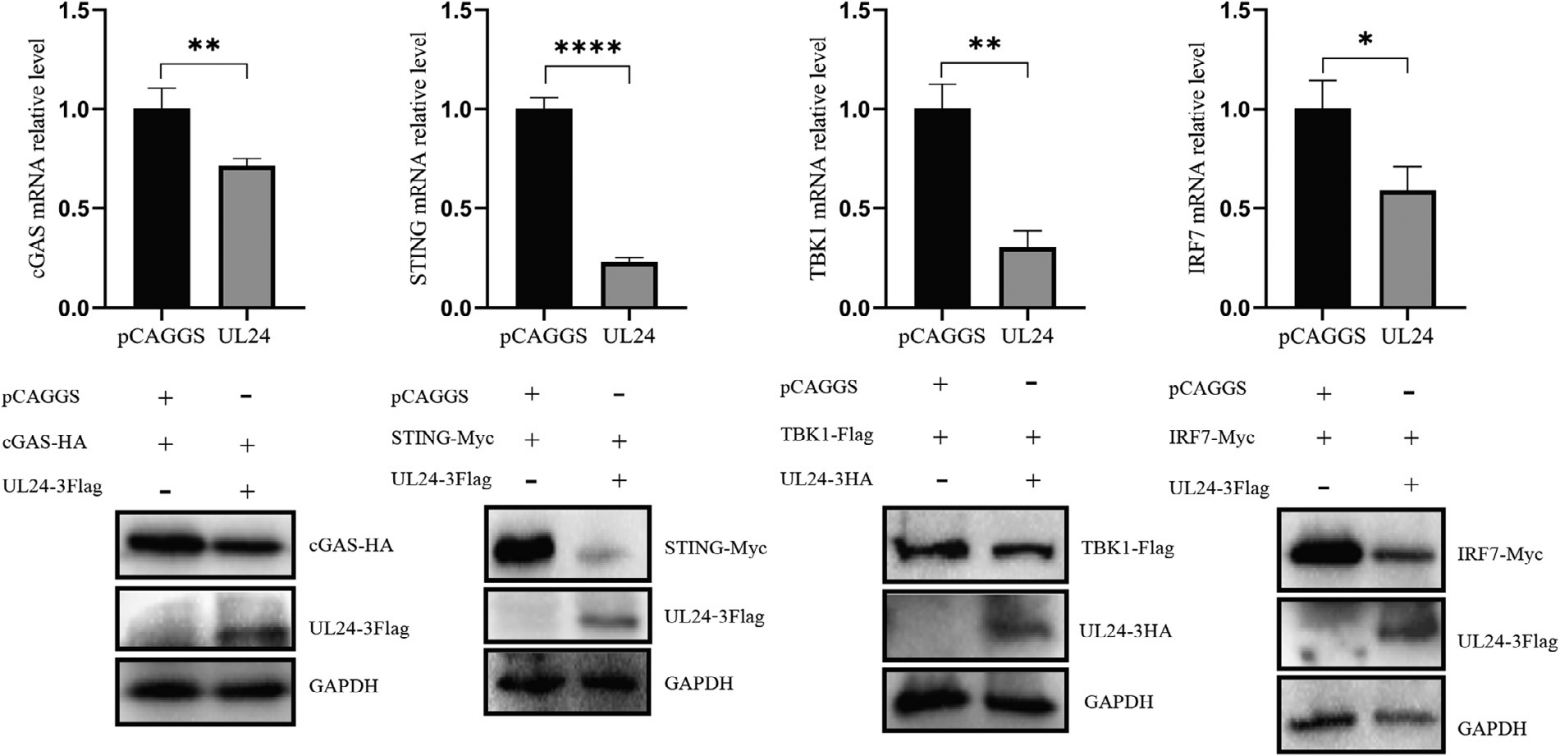
DPV UL24 downregulated the transcription of cGAS, STING, TBK1 and IRF7. UL24 and cGAS, STING, TBK1, and IRF7 eukaryotic expression plasmids were co-transfected into DEFs. After 36 h, RNA samples were collected, and the mRNA expression of cGAS, STING, TBK1 and IRF7 was measured using qPCR (*, *P* ≤ 0.5; **, *P* ≤ 0.01; ****, *P* ≤ 0.0001; and 1-way ANOVA).

Based on these results, UL24 can downregulate the expression of cGAS, STING, TBK1 and IRF7, and we used qPCR to measure the mRNA expression of these four proteins. The UL24 protein significantly downregulated the mRNA expression of cGAS, STING, TBK1 and IRF7 (Figure 5). These results indicate that one of the mechanisms by which DPV UL24 inhibits the host innate immune response is that the UL24 protein can downregulate the transcription of cGAS, STING, TBK1 and IRF7 and subsequently inhibit their expression; thus, signal transmission is blocked, and ultimately, IFN-β production is inhibited.

### DPV UL24 Initiates the K48-/K63-Linked Ubiquitination of IRF7

**Figure 6:**
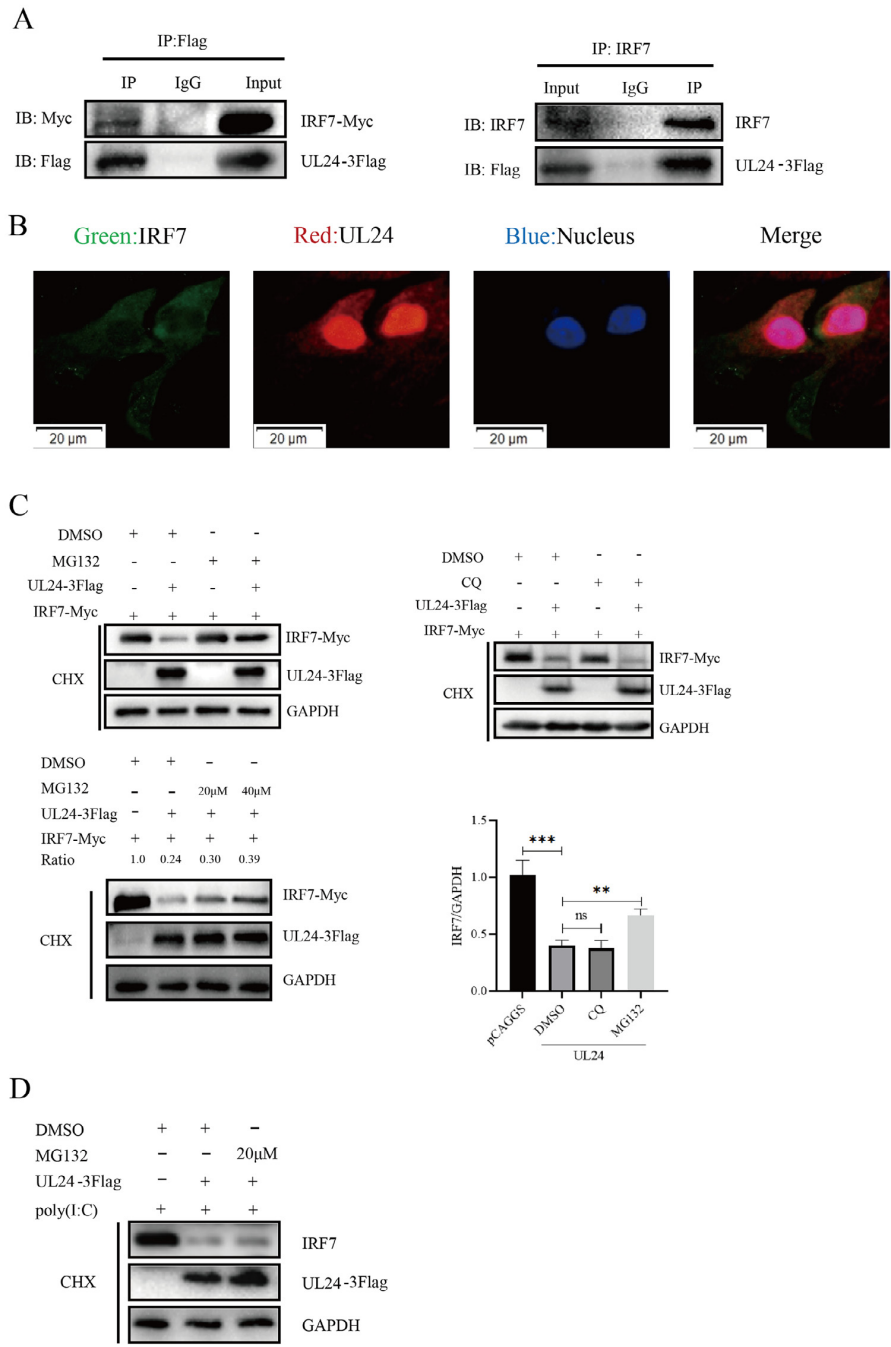
DPV UL24 promotes IRF7 degradation. (A) Interaction between the UL24 protein and IRF7. Left image: UL24-3Flag and IRF7-Myc were co-transfected into DEFs. After 36 h, the cell samples were collected and lysed, and Co-IP was performed with a mouse anti-Flag monoclonal antibody. Right image: UL24-3Flag was transfected into DEFs, the samples were collected, and Co-IP was performed with rabbit anti-IRF7. (B) The UL24 protein was colocalized with IRF7 in the cytoplasm. (C) The UL24 protein promotes IRF7 degradation. UL24-3Flag and IRF7-Myc were co-transfected into DEFs. After 24 h, the cells were treated with cycloheximide (CHX) and MG132 (40 μM) or CQ (200 mM) for 12 h. Cell lysates were collected, and their protein expression levels were measured. (D) MG132 inhibited the UL24 protein-mediated degradation of IRF7. UL24-3Flag was transfected into DEFs, after which the cells were stimulated with poly (I:C) to upregulate the expression of endogenous IRF7. The cells were then treated in the same way to determine the expression level of endogenous IRF7.

DPV UL24 can antagonize the expression of four node proteins in the cGAS-STING signaling pathway and downregulate IFN-β activity. Thus, we further explored the effect of the UL24 protein on IRF7. First, we confirmed that UL24 interacts with IRF7 and colocalizes with it in the cytoplasm (Figure 6A, 6B). To study whether the UL24 protein also affects the degradation of IRF7, we treated DEFs with the ubiquitin proteasome inhibitor MG132 or the autophagy lysosome inhibitor CQ. When protein synthesis was blocked by cycloheximide (**CHX**) treatment, the UL24-mediated decrease in IRF7 expression was alleviated in MG132-treated cells compared with that in the control cells, which not transfected with UL24-3Flag (Figure 6C). However, CQ did not influence the inhibitory effect of the UL24 protein on IRF7 expression. We further evaluated the effect of the UL24 protein on endogenous IRF7 expression in DEFs, and the results revealed that MG132 also suppressed the UL24-mediated downregulation of endogenous IRF7 expression (Figure 6D). These results indicate that the UL24 protein can promote the degradation of IRF7 through the proteasome pathway.

**Figure 7:**
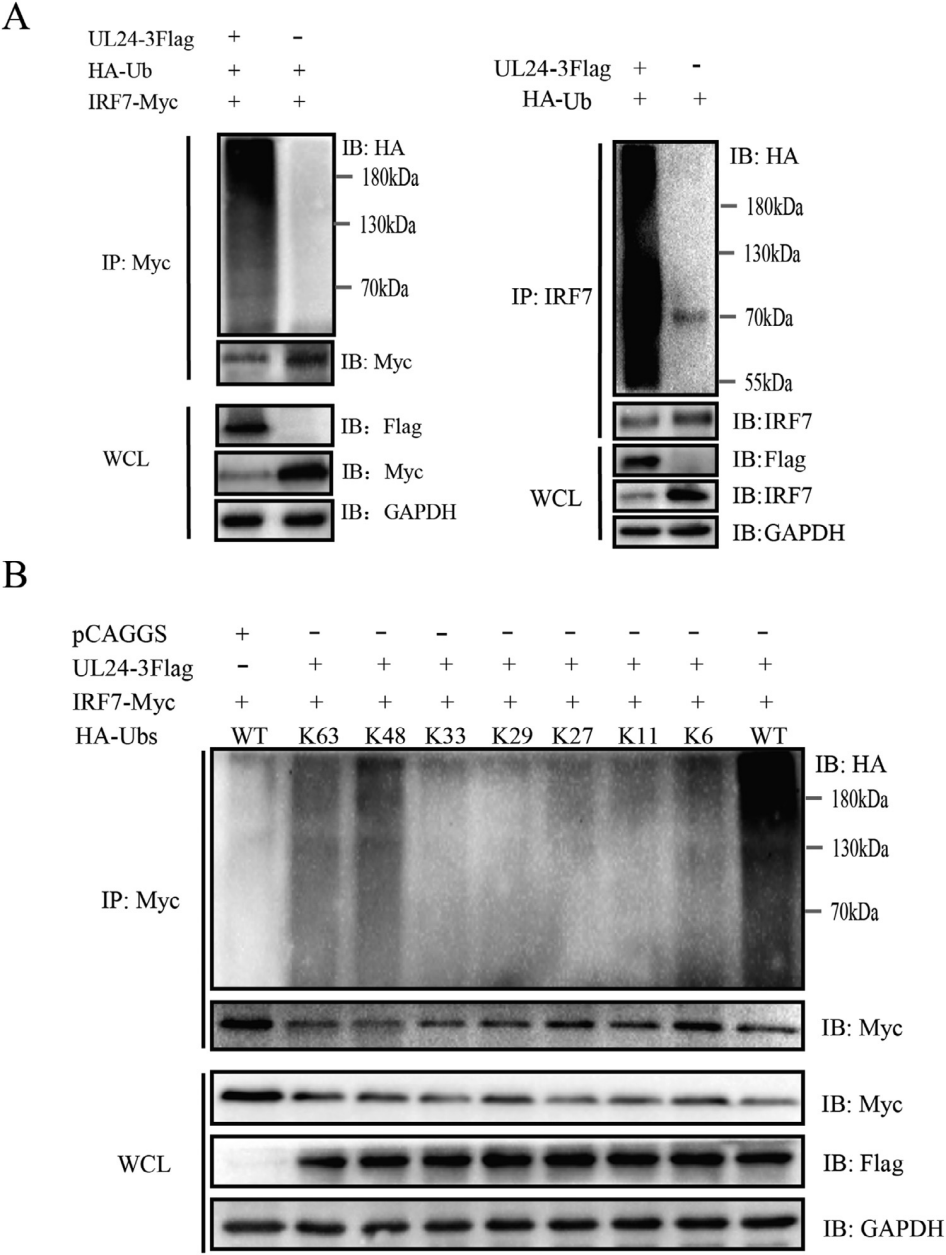
DPV UL24 initiates the K48-/K63-linked ubiquitination of IRF7. (A) The UL24 protein induces IRF7 ubiquitination. Left image: UL24-3Flag, IRF7-Myc and HA-Ub were co-transfected into DEFs, and the samples were collected 36 h later for Co-IP with a mouse anti-Myc antibody. Whole-cell lysates (WCLs) and coimmunoprecipitated complexes (IP:Myc) were analyzed by WB. Right image: UL24-3Flag and Ub-HA were co-transfected into DEFs for Co-IP with a rabbit anti-IRF7 antibody. (B) UL24-3Flag and IRF7-Myc were co-transfected with HA-Ub and its mutant plasmids into DEFs to detect the interaction between HA-Ub and IRF7-Myc.

We co-expressed UL24-3Flag, HA-Ub and IRF7-Myc in DEFs to determine whether the UL24 protein induces ubiquitination. Immunoprecipitation revealed that when UL24 was expressed, the level of ubiquitinated IRF7 was increased in DEFs (Figure 7A). The UL24 protein also induced the ubiquitination of endogenous IRF7 in DEFs (Figure 7A). Next, we investigated which type of ubiquitination was added to IRF7 when UL24 was expressed. Ubiquitin has seven lysine residues (K6, K11, K27, K29, K33, K48, and K63). Mutant plasmids retain only one lysine, and the remaining 6 lysines are mutated to arginine. We used plasmids to express the ubiquitin mutants to determine which type of ubiquitination was added to IRF7 when UL24 was expressed. Compared with those in the control group, when HA-Ub-K48 or HA-Ub-K63 was co-transfected with IRF7 and UL24, IRF7 exhibited obvious ubiquitination (Figure 7B). Therefore, UL24 mediated the K48-/K63-linked polyubiquitination of IRF7 to degrade it.

### The Conserved Domain of the UL24 Protein Regulates IRF7 Expression

**Figure 8:**
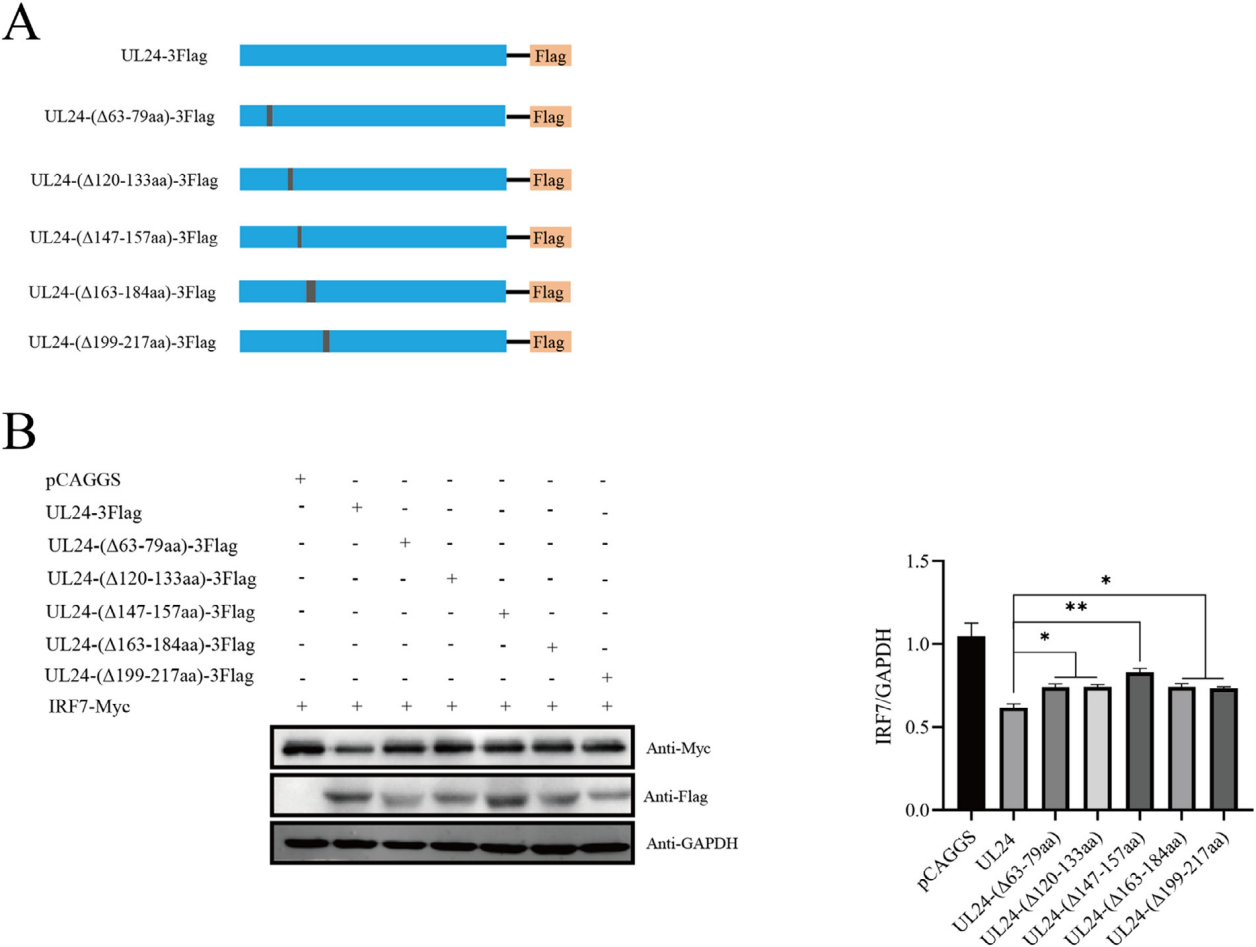
Effect of the DPV UL24 conserved domain on IRF7 expression. (A) Construction of the UL24 protein mutant plasmid. (B) UL24 and its mutant plasmids were co-expressed with IRF7 in DEFs, and the expression of IRF7 was detected at 36 h after transfection.

The UL24 protein has five highly conserved domains in herpesviruses. We compared the amino acid sequence of the DPV UL24 protein with the UL24-homologous amino acid sequence of the other three subfamilies of herpesviruses. According to the comparison results, we constructed five DPV UL24 protein domain deletion plasmids (Figure 8A). When the UL24 mutant plasmid was co-expressed with IRF7, the inhibitory effect of DPV UL24 on IRF7 expression was alleviated to varying degrees after the deletion of its conserved domain. When the UL24 protein 147 to 157 aa was deleted, the expression of IRF7 was most significantly restored (Figure 8B). Therefore, we speculate that amino acids 147 to 157 of the UL24 protein is the joint domain that inhibits the host's innate immune response.

## DISCUSSION

**Figure 9:**
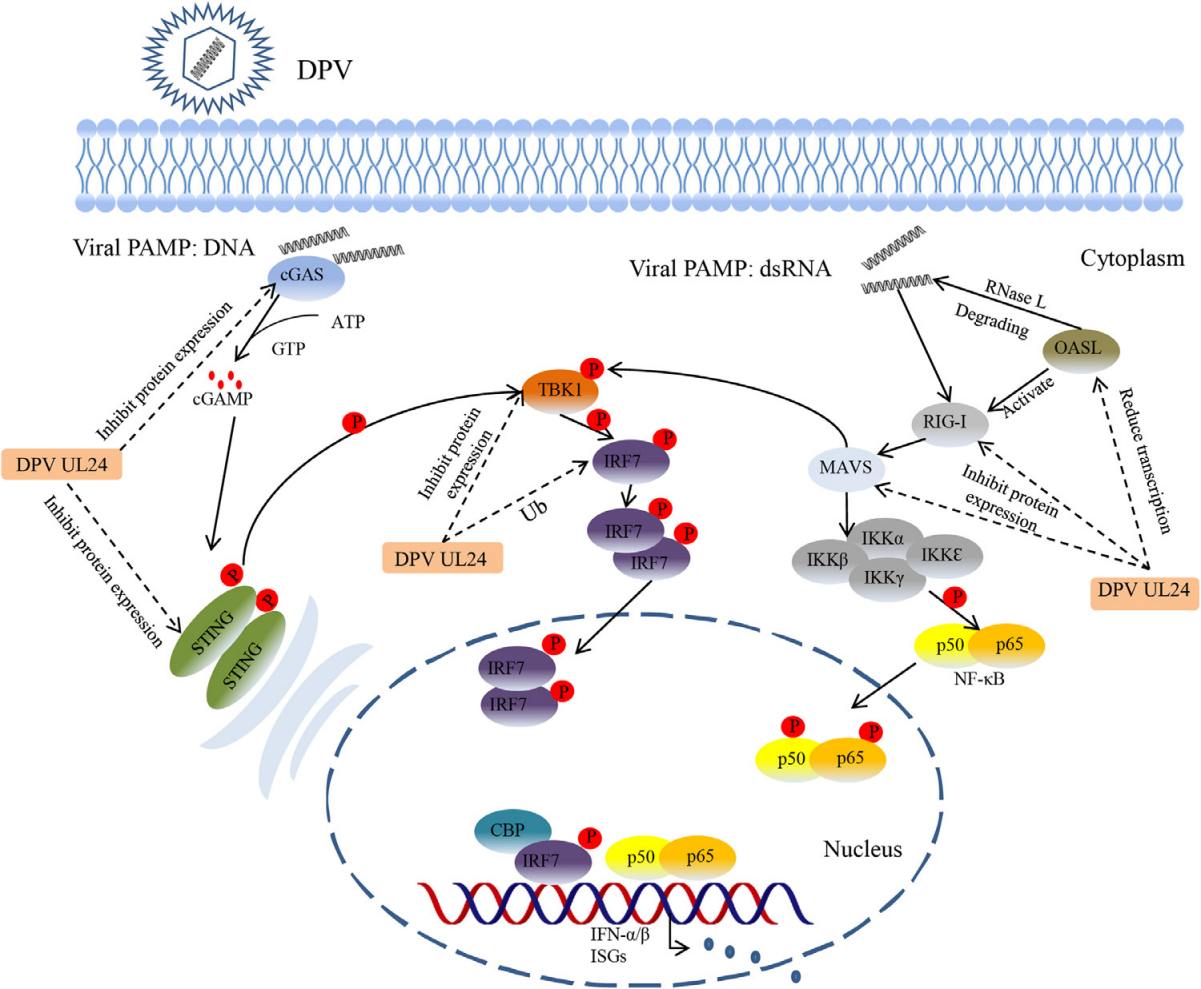
The mechanism by which DPV UL24 inhibits the host innate immune response. DPV UL24 inhibits the transcription of immune signaling molecules and promotes the ubiquitination and degradation of IRF7 to inhibit the host's innate immune response.

The innate immune system is the body's first line of defense against pathogen invasion, and type I interferons are key cytokines involved in early resistance to viral infection. To establish and maintain infection successfully, herpesviruses have evolved strategies, such as immune escape and latent infection, to evade the host's innate immune response to promote viral infection and replication (Zhu and Zheng, 2020, Zheng, 2018, White et al., 2012). Some progress has been made in research on whether the herpesvirus UL24 protein can antagonize the host innate immune response. HSV-1 UL24 can inhibit the activation of NF-κB by reducing the entry of the NF-κB subunits p65 and P50 into the nucleus (Xu et al., 2017), and PRV UL24 can downregulate the expression of p65 to inhibit the activation of IFN-β and NF-κB (Wang et al., 2020). It has been reported that DPV UL24 can inhibit the activity of the IFN-β promoter, but the effect of UL24 on host proteins in the immune signaling pathway has not been reported. In this study, we expressed the UL24 protein in DEFs and found that this protein significantly inhibited the innate immune response of host cells (Figure 9).

The cGAS-STING DNA signaling pathway plays a key role in host antiviral responses, and many herpesviruses have evolved various mechanisms to block this signaling pathway. Several studies have shown that viral proteins can inhibit cGAS-STING signal transduction; these proteins include HSV-1 VP22, which can downregulate cGAMP activity (Huang et al., 2018); HSV-1 VP16, which prevents IRF3 from binding to CBP (Xing et al., 2013); HSV-1 UL31, which promotes IRF3 degradation (Gong et al., 2022); HSV-1 ICP27, which targets TBK1 to reduce IRF3 activity (Christensen et al., 2016); and PRV UL13, which can hyperphosphorylate IRF3 and recruit RNF5 to promote STING ubiquitination (Kong et al., 2022, Bo et al., 2020, Lv et al., 2020). Studies on DPV have shown that its tegument proteins VP16, UL41 and Us3 can inhibit IFN-β activity and antagonize the host innate immune response (Li et al., 2020, Gao et al., 2022, Liu et al., 2022). In this study, we found that DPV UL24 inhibited not only DNA recognition signaling pathway-mediated IFN-β activation but also RNA recognition signaling pathway-mediated IFN-β activation. The mechanism by which DPV UL24 downregulates the expression of the cGAS, STING, RIG-I, MDA5, MAVS, TBK1 and IRF7 proteins may involve their ubiquitination-mediated degradation. In addition, the UL24 protein may also function as a potential endonuclease to degrade these mRNAs to downregulate their expression and inhibit the production of INF-β; this possibility needs to be explored in the future.

IRF3 is defective in avian cells, and IRF7 is necessary for initiating innate immune responses in avian cells during virus invasion (He et al., 2022, Xia et al., 2023, Lai et al., 2021, Huang et al., 2022, Huang et al., 2023). After viral infection, IRF7 is phosphorylated by TBK1, and it forms a homodimer and migrates to the nucleus, where it binds to the IFN-β promoter to initiate IFN-β transcription (Sun and Hornung, 2022, Gao et al., 2019, Lin et al., 2000). Therefore, viruses have evolved various strategies to inhibit IRF7 activation. In this study, we found not only that DPV UL24 can inhibit the transcription of IRF7 but also that UL24 can promote the degradation of IRF7 through the proteasome pathway. Therefore, this study added DPV UL24 to the extended family of viral proteins that promote IRF7 degradation through the ubiquitin-proteasome pathway.

A study on the influence of the UL24 protein on virus pathogenicity revealed that HSV-1 UL24, especially its conserved domain, which influences viral transmission to the host, is important for the virus to cause disease in the host (Leiva-Torres et al., 2010). When mice are infected with a UL24-knockout virus, the transmission of the virus to the trigeminal ganglion is blocked, which greatly reduces the viral titer in the trigeminal ganglion. The mice did not show clinical symptoms, and the latent infection and reactivation of the virus in the trigeminal ganglion were also greatly reduced (Jacobson et al., 1998, Rochette et al., 2015). Reduced pathogenicity was also observed in UL24 mutants of other herpesviruses, such as HSV-2 (Blakeney et al., 2005, Visalli et al., 2014). ORF37 of equine herpesvirus (**EHV-1**) and ORF35 of varicella-zoster virus (**VZV**) are homologous to the UL24 protein. EHV-1 did not produce neurotoxicity or lethal effects on mice after the deletion of ORF37 (Kasem et al., 2010). The deletion of ORF35 also reduces the pathogenicity of VZV (Ito et al., 2005). Whether the UL24 protein is involved in the pathogenesis of DPV remains to be explored.

Taken together, our results suggest that DPV UL24 is an effective inhibitor of IFN-β. DPV UL24 can downregulate the transcription of cGAS, STING, TBK1, and IRF7 and promote the degradation of IRF7 through the ubiquitin proteasome pathway, resulting in the inhibition of the host innate immune response.

## DISCLOSURES

All the authors declare that the research was conducted in the absence of any commercial or financial relationships that could be construed as potential conflicts of interest.
